# Developing, Implementing, and Evaluating a Care Management Model for Patients With Neuromuscular Diseases: Protocol for a Participatory Mixed Methods Study

**DOI:** 10.2196/82833

**Published:** 2026-02-24

**Authors:** Veronika Waldboth, Christina Schuler, Raffaella Willmann, Heidi Petry, Markus Weber, Barbara Grädel Messerli, Hannele Hediger, Iris Kramer, Gabriela Stettler-Nemecek, Martin Knoblauch, Georg Martin Stettner

**Affiliations:** 1 Department of Health Sciences Institute of Nursing ZHAW Zurich University of Applied Sciences Winterthur Switzerland; 2 Swiss Foundation for Research on Muscle Diseases Colombier Switzerland; 3 Centre for Clinical Nursing Science University Hospital Zurich Zurich Switzerland; 4 Neuromuscular Diseases Unit/ALS Clinic Health Ostschweiz (HOCH) Cantonal Hospital St. Gallen St. Gallen Switzerland; 5 Medical division for children and adolescents Bern University Hospital Bern Switzerland; 6 Swiss ALS Foundation Winterthur Switzerland; 7 Neuromuscular Center Zurich and Department of Pediatric Neurology University Children’s Hospital Zurich Zurich Switzerland

**Keywords:** neuromuscular diseases, rare diseases, care management, caregivers, quality of life, caregiver burden, mixed methods, study protocol, Switzerland

## Abstract

**Background:**

Neuromuscular diseases (NMDs) are often progressive conditions that can reduce quality of life and life expectancy and require long-term caregiving. As patients age, caregivers face increasing demands, often resulting in significant caregiver burden. Treatment and care are complex, and a coordinated, family-centered approach is indicated. The current care situation for individuals with NMDs and their families in Switzerland is poorly understood, and there is no standardized NMD care management available.

**Objective:**

The proposed study aims to assess the current care practices for individuals with NMDs in Switzerland, identifying unmet needs and challenges faced by patients, families, and health care providers. On the basis of these findings, a care management model will be developed, implemented, and evaluated to enhance support structures, strengthen specialized neuromuscular centers, and improve overall health care outcomes for affected individuals and their families.

**Methods:**

We planned a 3-phase participatory mixed methods study (the Care-NMD-CH study). First, qualitative descriptive data and quantitative survey data were used to assess the current state of care practices. Then, a care management model and training program were codeveloped based on these insights. Finally, the care management service was implemented in pilot specialized neuromuscular centers and evaluated using quantitative, qualitative, and integrated methods. In addition to interview data and patient records, we measured patient-reported outcomes (quality of life and self-efficacy), family-reported outcomes (family functioning and caregiver burden), and stakeholder-reported outcomes (quality of care and interprofessional collaboration). The study population comprises individuals with NMDs and their families living in Switzerland.

**Results:**

Ethics approval was obtained in 2020. Baseline data (T0) were collected and analyzed in 2021 (phase A). The NMD care management model (phase B) was developed based on these data and finalized in 2022. Following the start of implementation in 8 centers in 2023, data collection for process and outcome evaluation (T1-T4) commenced and was completed in May 2025. Data analysis is currently ongoing, and results will be published separately. This paper reports only the study protocol.

**Conclusions:**

This study represents an important step toward implementing and evaluating an evidence-based, family-centered care management service for individuals with NMDs and their families in specialized neuromuscular centers. Building on a 3-phase approach, the project identified gaps in current care; developed a structured care management model, including training; and evaluated its impact on patients, family members, and interprofessional care teams. Expected outcomes include possible improvements in the areas of care coordination, quality of life, and self-efficacy and family functioning. In addition, potential alleviation of disease and caregiver burden, as well as reduction in health care costs for those affected and the health system, will be investigated.

**International Registered Report Identifier (IRRID):**

DERR1-10.2196/82833

## Introduction

Many neuromuscular diseases (NMDs) follow a degenerative course. They can be associated with a high burden of disease, reduced quality of life, and shortened life expectancy [1,2]. However, owing to new disease-modifying therapies, improvements in symptomatic treatment, and advanced health technologies, NMDs now often have a better prognosis than ever before [3,4]. Despite these advancements, individuals with NMDs still require regular therapies as well as assistance in activities of daily living. In many families with affected children or adults, family members take over caregiving responsibilities [5-7]. As individuals with NMDs grow older, their caregivers must continue providing care for longer periods, which poses additional practical and psychosocial challenges [8,9]. The prolonged and intensive caregiving often leads to an increased caregiver burden [6,10,11]. Individuals with NMDs, their families, and the health system are often faced with considerable financial burdens [12-14].

Treatment and care of patients with NMDs is inherently complex and requires a coordinated, multidisciplinary, and interprofessional approach [15-17]. On the basis of disease stage, symptom burden, and personal priorities, the needs of individuals with NMDs and their family members can change constantly [17]. Therefore, families benefit from targeted support, assistance with care, care coordination, and family-strengthening interventions [5,7,18]. This is particularly relevant during health crises and important stages, such as the loss of ambulation, the affected young person’s transition into adulthood, and the end-of-life phase [6,19]. It is strongly advised to use a systemic approach when caring for the well-being of an individual with an NMD and their entire family.

Strengthening the support system for individuals living with these rare but often serious diseases is essential to ensure a sustainable care model for the future. Internationally recognized care guidelines exist for specific NMDs, emphasizing the necessity of multidisciplinary models that incorporate medical specialists and varied health care professional groups [20,21]. Individuals with NMDs benefit from a multidisciplinary approach through more comprehensive support, including clinical follow-up and resource use, improving their quality of life [22,23]. Care management services provided by health centers can support care coordination, improve the quality of life of frequent health care users, and enhance health care provider satisfaction, all while potentially reducing costs to the health care system [24,25]. Care management prioritizes interdisciplinary collaboration and effective communication among all the professionals involved in patient care to plan, implement, coordinate, evaluate, and prioritize services based on patient needs [26,27]. Through care management, individuals living with NMDs are empowered, and their self-efficacy and ability to self-manage their condition can improve [25,28,29]. The establishment and translation of evidence-based care management into practice would greatly benefit individuals with NMDs, as well as their families, by addressing their needs throughout all phases of care [30]. There is a need for an evidence-based, coordinated, multidisciplinary, and family-centered approach to care for the NMD population [20,30,31] to strengthen the support system for patients living with these rare and often severe conditions and create a sustainable care situation despite limited financial resources in the future.

However, in Switzerland, there is no standardized NMD care management available, and the current care situation and organization for individuals with NMDs is poorly understood. Given these circumstances, a comprehensive analysis and description of the current care practices for individuals with NMDs in Switzerland are crucial to identify effective strategies and areas for improvement.

The objectives of this study are to (1) describe the current state of care practices for individuals living with NMDs and identify unmet needs and challenges related to patients, their families, and the care team; (2) develop a care management model and training program for NMD care managers capable of addressing identified gaps in the support chain and enhancing the support offered by specialized neuromuscular centers; and (3) implement the standardized care management model and evaluate its impact on patients with NMDs and their families, on the interprofessional care team, and at the systems level.

## Methods

### Overview

This study uses a participatory mixed methods design that includes qualitative and quantitative data collection at different stages [32]. This design is suitable for studies that address health disparities through empowerment of marginalized or underrepresented populations, involve the target population in the research [32], and examine complex phenomena such as the evaluation of services in which various perspectives need to be included [33,34]. This study uses a pragmatic philosophical stance and a family systems theoretical framework [35]. Data integration occurs through methods (building and merging) and during interpretation (joint display and narrative) [32].

### Setting and Participants

Initial steps involved identifying the study cohort; establishing an expert panel (physicians, advanced practice nurses, and researchers) and a sounding board (affected individuals, family members, professionals, and other stakeholders) for feedback, guidance, and quality control; and creating a participant recruitment plan. The study cohort for the Care-NMD-CH study comprised patients with NMDs and their families living in Switzerland. In Switzerland, 7 neuromuscular centers provide specialized outpatient and inpatient care for individuals with NMDs. These centers are hospital-based tertiary national reference centers accredited by the National Coordination of Rare Diseases. Four groups of study participants were identified: (1) patients with NMDs, (2) family members (parents, siblings, partners, and other relatives), (3) professionals who are involved in the care, and (4) other stakeholders (eg, nurses, physiotherapists, and members of patient organizations). The study included participants from all linguistic regions of Switzerland. Recruitment included the identification of collaborating institutions and gatekeepers, who informed potential participants about study participation.

### Study Phases

#### Overview

**Figure 1:**
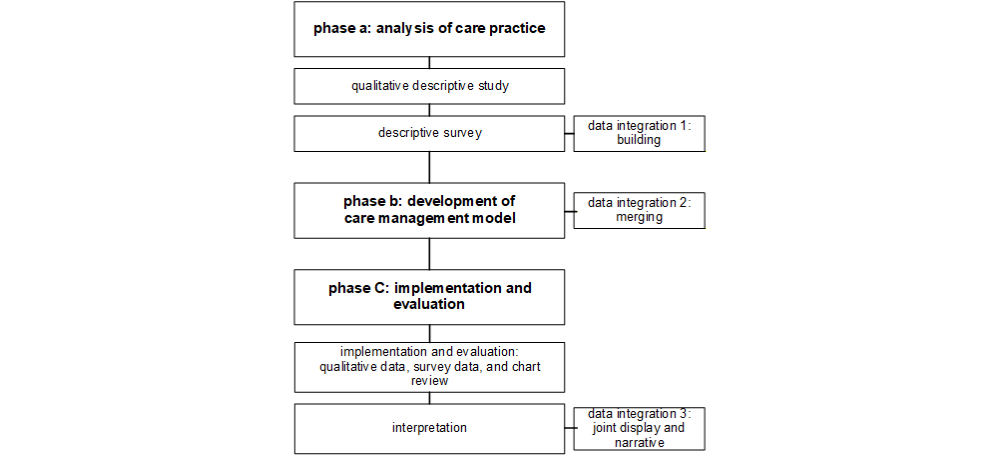
Overview of the study phases.

According to the study aims, there were 3 phases (A-C) over a duration of 5 years (2020-2025; Figure 1). In phase A, qualitative and quantitative data were collected using instruments tailored to the needs of the individuals being studied to assess the current state of care practice [36]. In phase B, a new care management model and training program for NMD care managers was developed involving relevant stakeholders, including patients with NMDs, their families, and health care professionals. In phase C, the new care management model was implemented in pilot specialized neuromuscular health centers, and processes and outcomes were evaluated using a pretest and posttest design. The following criteria were used to reflect on the quality of the study: rigor, credibility, and transferability [37-39].

#### Study Phase A: Analysis of the Current State of Care Practice

##### Qualitative Descriptive Study

At first, a qualitative descriptive research design was applied to explore and describe the current state of care practice [40,41]. This approach facilitates close engagement with the data and enables a subjective exploration of people’s everyday experiences and needs [42,43]. A purposive sampling strategy was used to recruit participants who met the inclusion criteria and were able to provide in-depth information addressing the research question [44]. Data were collected through one-on-one and focus group interviews with patients with NMDs, family members, professionals who were involved in their care, and other stakeholders (planned: n=45). For ethical reasons, patients and family members participating in interviews were aged ≥8 years.

Separate semistructured interview guides were developed for each participant group containing age-appropriate open-ended and probing questions. The questions assessed the general care situation, benefits of care, gaps in the support chain, unmet needs, experiences within the care network, barriers to sustainability, communication, and collaboration. Audio data were transcribed verbatim according to the guidelines set by Dresing and Pehl [45]. The latest version of MAXQDA (VERBI GmbH) was used for data analysis [46]. The transcripts were analyzed using the qualitative data analysis principles outlined by Saldaña [47]. The inductive analysis process involved four steps: (1) open coding without using prior concepts or predefined categories, (2) code allocation into main areas, (3) bundling into subcategories, and (4) comparison and revision of subcategories and generation of categories. The sociodemographic data of participants were collected and analyzed descriptively.

##### Descriptive Survey

The quantitative part of phase A consisted of an online descriptive survey to collect further information on the current practices of care from a larger group of participants. The results from the qualitative study were used to develop the survey components. This was the first data integration point in our mixed methods study, where results from one data collection phase (qualitative) informed the next data collection phase (quantitative), an integration approach referred to as “building” [32,36].

##### Quantitative Data Collection

The online survey included self-developed questions based on the obtained qualitative results and standardized instruments (Table 1). Four questionnaires were developed, one for each group of participants (patients, family members, professionals, and other stakeholders; planned sample: n=500). Patients and family members who were expected to complete the online survey independently were aged ≥14 years. The questionnaires consisted of Likert-type questions, visual analog scales, and closed- and open-ended questions. Topics common to all participant groups included sociodemographics, information about the care situation, satisfaction with care, gaps in the support network or services, unmet needs, experiences within the care network, barriers to sustainability, and collaboration. Group-specific measures included the primary end point (quality of life for patients) and the following secondary end points: self-efficacy for patients, family functioning and caregiver burden for family members, and quality of care and interprofessional collaboration for professionals. The survey was conducted and managed using the web-based REDCap (Research Electronic Data Capture; Vanderbilt University) tool [48]. The survey was made available online, and participants were recruited using a multitiered approach to gather a range of viewpoints. To ensure anonymization of data and to be able to link data over different time points, each participant marked the questionnaire with a personal code developed according to a defined scheme.

**Table 1:** Overview of survey components.

	Patients with NMDs^a^	Family members	Professionals	Other stakeholders
Component 1	Self-developed questions based on the qualitative study	Self-developed questions based on the qualitative study	Self-developed questions based on the qualitative study	Self-developed questions based on the qualitative study
Component 2	Health-related quality of life (WHOQOL-BREF^b^ [49])	Family functioning (FAD^c^ [50])	Quality of care (single-item measure [51])	Acute events or hospitalization, morbidity, and mortality (documents and statistics)
Component 3	Self-efficacy (GSE^d^ [52])	Caregiver burden (BSFC^e^–Short Version [53])	Interprofessional collaboration (IPC^f^ [54])	Costs, financing, and reimbursement (documents and statistics)

^a^NMD: neuromuscular disease.

^b^WHOQOL-BREF: World Health Organization Quality of Life Scale–Brief.

^c^FAD: Family Assessment Device.

^d^GSE: General Self-Efficacy Scale.

^e^BSFC: Burden Scale for Family Caregivers.

^f^IPC: interprofessional collaboration.

##### Quantitative Data Analysis

The data analysis will be carried out using the latest version of SPSS Statistics (IBM Corp) [55]. The dataset will be examined for missing and out-of-range values using frequency tables, and imputation procedures will be applied according to missing value mechanisms. Statistical assumptions will be assessed before analysis, and the choice of tests will be adapted as appropriate based on the data characteristics and the actual sample. We will describe the sample using frequencies, percentages, means, SDs, and ranges. The instrument’s internal consistency, measured via the Cronbach α, must be above 0.80. Minimum values of 0.60 can be accepted for small sample sizes and scales with few items. The significance level will be set at <.05. Quantitative findings will be discussed with the expert panel and sounding board to check for clinical relevance and credibility.

#### Study Phase B: Development of a Care Management Model

The care management model was developed following the structure of a logic model [56]. Therefore, the qualitative and quantitative results from phase A were integrated to delineate unmet needs and challenges for the participant groups, which can be addressed by NMD care managers. This was the second data integration point in our mixed methods study, where integration of data occurred for comparison through merging quantitative and qualitative findings from phase A [32,36]. The developed model outlined the roles and responsibilities of NMD care managers and contained a comprehensive requirement and competency profile that covered both pediatric and adult care. Experts from the expert panel and sounding board were involved in the development of the model following a creative cyclical process of repeated phases of reflection, planning, action, observation, reflection, and replanning [57].

On the basis of the developed NMD care management model, a training program for NMD care managers was developed, which consisted of classroom and online learning sessions and covered a broad spectrum of essential topics related to NMD-specific patient care. Key areas included pathophysiology, diagnostics, supportive interventions, and nursing and treatment options; psychosocial aspects, palliative care, and the relevant transitions (eg, from pediatric to adult care); and systemic counseling, interprofessional collaboration, and leadership. The training program incorporated existing standards of care for individual NMDs as well as care priorities and counseling topics that are shared across diagnoses. NMD care managers were expected to network, complete an internship, and prepare a case report. The 2-year part-time training program started with the implementation of the care management model in the pilot specialized health centers.

#### Study Phase C: Implementation and Evaluation

##### Implementation

Phase C comprised the recruitment of pilot specialized neuromuscular health centers for the 2-year implementation and a context analysis ahead of the implementation. The context analysis was necessary to better understand the specific institutional situation, overcome implementation challenges, and identify factors that may influence intervention uptake and study outcomes [58,59]. The context analysis was based on the Integrated Promoting Action on Research Implementation in Health Services framework [58,60]. The analysis was performed through focus group interviews (planned: n=8) using a semistructured interview guide based on the Integrated Promoting Action on Research Implementation in Health Services implementation framework, which focuses on recipients, evidence, and inner (local) and outer (organization) context [60]. The aim was to identify barriers to and facilitators of adapting the NMD care management model to individual institutional needs. Interviews were audio recorded and transcribed verbatim and analyzed as outlined in phase A.

##### Evaluation

The process and outcome evaluation was performed using formative and summative evaluation strategies. Formative evaluation assesses the local implementation strategy, monitors the process, and allows for real-time adjustments, improving the chances of success [61,62]. This was achieved through the training program, regular contact between the clinical team and research team, and documentation of clinical practice. After each contact with patients and their families or any other activity related to their role, this information was documented by the NMD care manager and collected by the research team for further analysis (care manager record using a case report form). The record included anonymized data, such as the type of contact, counseling topics, assessment, and quality of life. Summative evaluation of the NMD care management model was conducted by collecting qualitative and quantitative data through interviews, surveys, and the care manager record on 4 levels and at different time points (T1-T4; Table 2).

**Table 2:** Overview of data collection across time points.

Participants	Phase A (2020-2021)-T0 (baseline)	Phase B (2022)	Phase C (2023-2025)			
		Context analysis	T1 (6 months)	T2 (12 months)	T3 (18 months)	T4 (24 months)
Patients with NMDs^a^	Interview and survey	—^b^	—	Survey and care manager record	Interview	Survey and care manager record
Family members	Interview and survey	—	—	Survey	Interview	Survey
Professionals	Interview and survey	Interview	Interview	Survey	—	Survey
Stakeholders	Interview and survey	—	—	Survey	—	Survey

^a^NMD: neuromuscular disease.

^b^Not applicable.

##### Qualitative Descriptive Study, Data Collection, and Analysis

Six months after the start of implementation (T1), focus group interviews were conducted with professionals involved in the care at the pilot specialized neuromuscular health centers to evaluate the implementation and impact of the NMD care management model (planned: n=8). At 18 months after implementation (T3), semistructured interviews were undertaken with patients and their families to assess the potential benefits of the care management services (planned: n=16). The semistructured interview guides, data transcription, and analysis corresponded to those applied for the qualitative study in phase A.

##### Quantitative Data Collection and Analysis

The survey described in phase A was repeated in phase C under the same conditions: the same quantitative data were collected at 12 (T2) and 24 months (T4) after the start of implementation.

The T2 and T4 samples will be described using descriptive statistics (frequencies, percentages, means, SDs, and ranges). Changes over time (T0, T1, and T2) will be examined using inferential statistics and linear mixed models. Statistical assumptions will be assessed before analysis, and the choice of tests will be adapted as appropriate based on the data characteristics and the actual sample. A minimum sample size of 24 patients was estimated using a simulation-based approach to detect a medium effect, accounting for attrition in repeated-measure analyses of the primary end point. Quantitative findings will be triangulated with qualitative results to explain observed patterns, and not significant changes will be discussed with the expert panel and sounding board to determine whether they are still clinically meaningful and inform refinement of the care management model and its implementation strategy.

##### Data Integration During Interpretation

Finally, findings will be integrated through joint displays and narrative at a third data integration point [32]. Therefore, both qualitative and quantitative findings will be reported together on a theme-by-theme basis for selected themes (weaving approach). Quantitative data will present the general trends and patterns, whereas qualitative data will be used to illustrate and deepen understanding of those trends through participant narratives. This integrated approach will be used in reporting and presentations to ensure a coherent and holistic account of the study’s outcomes. Additionally, data will be brought together through a joint display using a table or matrix to draw out new insights beyond the information gained separately [32]. Therefore, a triangulation protocol will be applied.

### Ethical Considerations

This study adheres to Swiss national laws and the ethical principles of the Declaration of Helsinki [55]. Ethics approval was received from the lead ethics committee in Ethikkommission Zürich (Business Administration System for Ethics Committees ID 2020-01882) and the ethics committees of the relevant study sites (Kantonale Ethikkommission Bern, Ethikkommission Nordwest- und Zentralschweiz EKNZ, Commission cantonale d'éthique de la recherche sur l'être humain CER-VD, Ethikkommission Ostschweiz EKOS, Ethikkommission Tessin). This study was classified as human research with the exception of clinical trials (Human Research Ordinance; chapter 2), risk category A (low risk), and registered in the Registry of All Projects in Switzerland (2020-01882). Participants provided informed consent before inclusion in the study. All participants were informed that their participation was voluntary and that they could withdraw from the study at any time. For data security, consent forms and other documents containing identifying information will be stored in a restricted archive for 10 years after the study ends. No compensation was provided to participants.

## Results

This study received ethics approval in 2020. It was funded in 3 tranches in 2020, 2022, and 2024. Baseline data on the current state of care practices (T0) were collected in 2021 (phase A). A total of 52 interviews with individuals with NMDs or caregivers and health care professionals were conducted. In total, 360 people took part in the online survey. In 2022, based on the data from phase A, a care management model was codeveloped (phase B), which will be reported elsewhere. In phase C, the care management model was implemented in 8 pilot neuromuscular centers in 2023 with a concurrent training program of 150 training hours. Subsequently, data collection continued until May 2025, where data were collected at different time points (T1-T4) to evaluate the possible effects of implementing the care model. In phase C, a total of 24 interviews were conducted, and 409 people took part in the online survey. Data analysis and integration in this phase are currently ongoing, and findings will be published separately. This publication reports on the study protocol.

## Discussion

### Expected Findings

The proposed study investigates the current care situation for individuals with NMDs and their families in Switzerland and aims to develop, implement, and evaluate a standardized NMD care management model targeted at this population. The study’s objective is to improve clinical outcomes, patient and family well-being, and the efficiency of health care services. Expected outcomes include enhanced patient and caregiver satisfaction through more coordinated, family-centered care. Codeveloping an NMD care management model with patients, family members, and professionals is expected to result in a comprehensive model and targeted implementation strategies. This model will meet real-life needs, with the potential to significantly improve health-related quality of life, self-efficacy, and family functioning while simultaneously reducing caregiver burden and the frequency of acute health care events such as hospitalizations. Strengthening collaboration among health care professionals within specialized neuromuscular centers and with external institutions through effective information exchange and optimized resource use can reduce overall health care costs and support a more sustainable model of care for patients with NMD. Additionally, this study aims to build and strengthen a network of specialized NMD care managers within and across health care settings, facilitate education and the exchange of NMD expertise, establish a standardized NMD care management model, and develop a training program for NMD care managers. This participatory mixed methods protocol developed for the Care-NMD-CH study can be adapted and applied to other settings and patient populations with complex health care needs, providing a scalable and generalizable framework for improving care management practices. This project represents the first time in Switzerland that the care of patients with NMDs, in terms of both the quality and quantity of services, is being examined.

### Strengths and Limitations

This study targets an underserved population and introduces a framework for the development and implementation of care management services. The needs analysis and cocreation conducted in phases A and B with relevant stakeholders can enhance the credibility of the findings and may positively influence the acceptability and long-term sustainability of the care management model. Methodological choices and a mixed methods design in process and outcome evaluation (phase C), drawing on multiple data sources, enable triangulation across patient, family, professional, and system levels and support methodological rigor.

There are some potential limitations that should be noted at this stage. We recruited from different study sites in Switzerland, all of which have their own subcultures, focused specializations and interests, and differences in internal and external processes. A comparable level of diversity among participants may lead to heterogeneous results, and common themes in our data may be difficult to identify. However, it is important to recruit study sites and participants from across Switzerland to better understand the current practices with regard to this group of rare disorders and the impact on the multifaceted quality of patient care in the country. Another potential challenge is that only a specific group of participants, such as those with a higher educational level, greater familiarity with digital media, and a lower disease burden, participated in the study. In addition, study sites involved in participant recruitment may have preferentially contacted individuals for interviews who were perceived as more suitable, which could introduce selection bias. Language may pose a challenge during the training course for new care managers, as many Swiss residents do not speak all three main official languages, and the English proficiency of potential participants may be inconsistent. The quantitative evaluation relies on pretest-posttest comparisons without a control group, limiting causal inference. The range of assessed outcomes can diminish the interpretability of the findings, and analysis is pending, so conclusions about broader impact remain preliminary.

### Conclusions

The introduction of an evidence-based and family-centered care management model in pilot specialized neuromuscular centers will have a multifaceted impact on the care and treatment of this vulnerable patient group. It is expected that a standardized NMD care management model will strengthen the support system for patients living with these rare but severe conditions and create a sustainable care situation despite limited financial resources in the future. The Care-NMD-CH project represents an important step toward a comprehensive family-centered care management model for individuals with NMDs, which can be transferred to other chronic and rare disorders using this study framework. This study will advance care for individuals with NMDs and their families by identifying gaps in current practices; developing a targeted NMD care management model with training; and evaluating its impact on patients, families, and care teams. The findings will guide coordinated, family-centered, and sustainable care, offering a structure to improve clinical practice, patient and family experience, team collaboration, and health system support.

## Abbreviations

NMD: neuromuscular disease
REDCap: Research Electronic Data Capture

## Data Availability

The datasets generated or analyzed during this study are not publicly available due to ethical restrictions. Instruments and templates are available from the corresponding author upon reasonable request.
